# Scientific epistemology beliefs and acceptance of Traditional Chinese Medicine: A multigroup analysis based on the UTAUT model in Southern China

**DOI:** 10.1016/j.heliyon.2024.e33136

**Published:** 2024-06-17

**Authors:** Minrui Zhang, Aiyuan Cai, Kexin Jin, Jiaying Huang, Dan Li, Meihui He, Ruixiang Gao

**Affiliations:** a Department of Pediatrics, Guangdong Provincial Hospital of Traditional Chinese Medicine, Guangzhou, 510120, China; b The Second Clinical Medical College, Guangzhou University of Chinese Medicine, Guangzhou, 510405, China; c Shenzhen Zhongshan Obstetrics & Gynecology Hospital, Shenzhen, 518100, China; d Xiaorong Luo's Renowned Expert Inheritance Studio, Guangdong Provincial Hospital of Traditional Chinese Medicine, Guangzhou, 510120, China; e Yue Bei People's Hospital, Shaoguan, 512026, China; f Center for Studies of Psychological Application, Guangdong Key Laboratory of Mental Health and Cognitive Science, and School of Psychology, South China Normal University, Guangzhou, 510631, China

**Keywords:** Pediatric TCM, Unified theory of acceptance and use of technology, Scientific literacy, Multigroup path analysis

## Abstract

**Purpose:**

This study for the first time delves into the intricate relationship between scientific literacy and the acceptance of traditional Chinese medicine (TCM) by employing a multigroup path analysis based on the Unified Theory of Acceptance and Use of Technology (UTAUT) model. We adopted Scientific Epistemology Belief (SEB) as an indicator for measuring scientific literacy due to its comprehensive reflection of individuals' understanding of scientific knowledge and knowing. In assessing TCM acceptance, we focused on Chinese parents' receptivity towards pediatric TCM, as it offers a more genuine representation of actual inclinations.

**Methods:**

A convenience sample of 1016 Chinese parents in Southern China was assessed using online Likert-scale questionnaires on SEB and UTAUT determinants (including performance expectancy, social influence, risk awareness, and facilitating conditions). A K-means cluster analysis was employed to discern distinct SEB profiles, followed by a multigroup path analysis to ascertain UTAUT model variations across these profiles.

**Results:**

Five SEB profiles were identified, namely, intermediate, absolutistic, multiplistic, sophisticated, and evidence-based. Evidence-based believers manifested the highest pediatric TCM acceptance, albeit with elements of blind faith, while multiplistic skeptics, prone to questioning everything, displayed the least acceptance. The absolutistic, intermediate, and sophisticated demonstrated moderate TCM acceptance levels, with the intermediate profile outscoring both absolutistic and sophisticated. These findings highlight that individuals with high scientific literacy do not blindly endorse TCM, nor do those with limited scientific understanding fully appreciate TCM's merits.

**Conclusion:**

SEB significantly moderates TCM acceptance factors in the UTAUT model, indicating that extremes in scientific knowledge spectrum result in less balanced TCM perspectives. Our findings pave the way for novel insights into harmonizing modern and traditional medical practices.

## Highlights

• Nonlinear relation between scientific literacy and TCM acceptance was first observed.
• The multigroup UTAUT model yielded significant results.
• Evidence-based parents exhibited the greatest pediatric TCM acceptance.
• Multiplistic skeptics showed the least acceptance.
• The intermediate surpassed both the absolutistic and sophisticated in acceptance.

## 1 Introduction

Traditional Chinese medicine (TCM) has garnered renewed global interest following claims of health benefits against COVID-19 [1]. However, integrating TCM into mainstream modern medicine faces challenges. The major difficulty is the relatively low quality of TCM research, making the development of rigorous scientific approaches for TCM evaluation extremely crucial [2]. For instance, the Chinese government reported an apparent cure rate of Qingfei Paidu decoction against COVID-19 exceeding 90 % (661/701), but this is based only on uncontrolled data statistics, lacking placebo-controlled, double-blind randomized controlled trials or further systematic reviews for verification [3]. Interestingly, attitudes towards TCM diverge between China and the West. In Western nations, where modern science originated [4], TCM is popularly embraced as a complementary or alternative medicine [5]. In contrast, Chinese people with a long history of using TCM seem conflicted—appreciating traditional remedies while also questioning TCM's benefits compared to scientifically tested modern medicine [6].

However, contrary to the prevalent belief that TCM primarily appeals to the elderly, those with limited modern scientific and medical knowledge, or residents of economically disadvantaged areas, a recent data mining analysis of a decade of real-world medical visits in China has revealed a surprising trend. Young adults aged 25 to 35 now represent the largest demographic seeking TCM treatments, outnumbering the elderly. Additionally, a significant increase in TCM patients is observed among children aged 0 to 8, a trend attributed to their parents—predominantly within the 25–35 age bracket—taking them for TCM consultations. These findings challenge the stereotype of TCM's primary users and advocates in health management and well-being maintenance [7]. Nevertheless, few studies have investigated the reasons behind this demographic's increased trust in and reliance on TCM. Among the potential factors including scientific literacy, medical knowledge, social influence, exposure to TCM information, perceptions of TCM's effectiveness and risks, the key determinant remains unidentified and calls for more research. Some questionnaire surveys suggest that this trend could be due to this demographic's increased exposure to TCM promotional campaigns and popularization efforts [[8], [9], [10]]. Notably, the relationship between scientific literacy and the acceptance or adoption of TCM practices in this demographic has not yet been explored. For these highly educated young people, the question of whether modern science, which originated in the West, conflicts with traditional practices rooted in China's long history, presents a fascinating area for further investigation.

Moreover, most existing studies on the factors influencing TCM acceptance [[8], [9], [10]] lack an established theoretical framework, resulting in fragmented variable selection and limiting their impact within the academic community. This highlights the necessity for a more structured approach in researching TCM acceptance. In this pioneering study, we utilized two theoretical frameworks—the Unified Theory of Acceptance and Use of Technology (UTAUT) model and Scientific Epistemic Beliefs (SEB)—to explore the relationship between scientific literacy and TCM acceptance. The UTAUT model integrates crucial variables such as peer influence, exposure to TCM information and facilities, as well as perceptions of TCM's effectiveness and risks. SEB, on the other hand, provides insights into a deep understanding of scientific knowledge and discovery. By investigating the moderating effects of SEB on the UTAUT model, we aim to gain nuanced insights into why highly educated young people demonstrate a greater trust in TCM. As this demographic increasingly becomes a significant influence and holds greater decision-making power in society, their endorsement and support for TCM will directly impact the future development and integration of TCM into the realm of modern medicine.

### 1.1 UTAUT

First introduced by Venkatesh and colleagues [11], the UTAUT model integrates earlier technology adoption models into a unified framework predicting user acceptance and usage behavior. UTAUT proposes four key determinants: performance expectancy, effort expectancy, social influence, and facilitating condition. Performance expectancy refers to the degree an individual believes using the technology will improve outcomes. Effort expectancy represents perceived ease of use. Social influence encompasses others' opinions, social norms and persuasion. Facilitating condition includes technical and organizational support structures. In the model, performance expectancy, effort expectancy, social influence, and facilitating conditions influence technology usage behavior through usage intention, while facilitating conditions also directly impacts usage behavior. Factors such as gender, age, usage experience, and voluntariness often play a moderating role in the model [12].

Numerous healthcare studies demonstrate UTAUT's ability to explain adoption of telemedicine, mHealth, e-mental health, and other innovations. Cao et al. found trust, performance expectancy, and effort expectancy positively predicted mHealth app usage intention among young Japanese adults [13]. Damerau et al. [14] revealed mental health status and gender significantly influenced e-mental health intervention acceptance among diabetes patients. Chang [15] identified health consciousness and trust as key drivers of wearable medical device adoption intentions in Taiwan. A survey of Filipino neurologists showed performance expectancy and facilitating condition were the strongest predictors of teleneurology uptake [16]. However, no research to date has applied the UTAUT model to TCM treatment. Besides, it is noteworthy that many of these studies considered the additional factor of trust beyond the classic UTAUT variables, as medicines, while providing therapeutic benefits, also carry risks of side effects, ranging from minor symptoms (such as diarrhea, nausea, or dizziness) to severe reactions (such as shock, renal failure, or cancer) [17]. Users' trust is thus an important factor in healthcare technology acceptance.

**Fig. 1:**
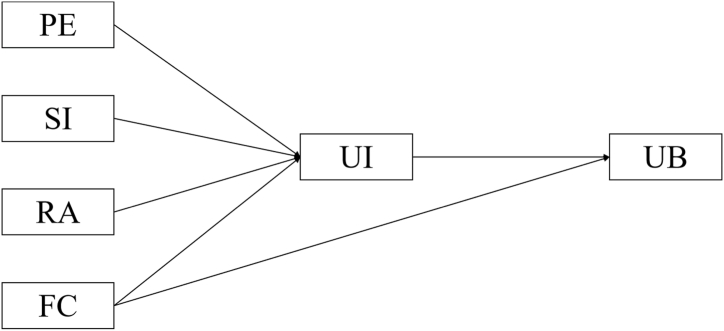
The UTAUT model of TCM. PE = performance expectancy, SI = social influence, RA = risk awareness, FC = facilitating condition, UI = usage intention, UB = usage behavior.

In this study, we did not utilize trust, but opted instead for the related construct of risk awareness to reflect users' safety perceptions of TCM. Research indicates trust and risk awareness are two significant variables in attracting and retaining users [18]. Compared to western medicine, TCM encounters greater trust issues among patients, likely due to perceptions of higher risks given the lack of rigorous scientific evidence verifying its benefits and potential harms [6]. We posit that introducing risk awareness into the UTAUT model can more accurately capture the role of safety concerns in influencing usage intention and behavior. Furthermore, since the majority of people receive TCM treatments from doctors rather than administrating TCM themselves [19], measuring ease of use and effort is deemed unnecessary in our study; thus, effort expectancy is excluded from the model (refer to Fig. 1 for the complete diagram).

### 1.2 SEB

In UTAUT, key variables on usage intention and behavior are typically affected by moderators such as gender, age, experience, and voluntariness [11]. However, an analysis of 10 years' Chinese medical data suggests individuals aged 25–35 with modern science education constitute the primary demographic seeking TCM treatments [7]. Beyond age and experience, scientific literacy thus emerges as a potential key influencer.

Scientific literacy involves utilizing scientific knowledge and skills for explaining phenomena, problem-solving, and decision making [20]. It encompasses content knowledge, procedural skills, and epistemic beliefs [21]. A broader health and environmental concepts enables individuals to interpret conflicting information more effectively [22]. Evidence suggests that the core of scientific literacy lies in SEB.

SEB pertains to the conviction about the nature of knowledge and knowing in science. A prominent framework proposes four key dimensions of SEB: source, certainty, development, and justification [23]. The source dimension reflects beliefs about where scientific knowledge originates and who controls it. The certainty dimension represents beliefs regarding how stable or certain scientific knowledge is. The development dimension refers to views on whether science is unchanging or constantly evolving. The justification dimension involves how individuals evaluate scientific claims using evidence. Research employing a person-centered approach (e.g., latent profile analysis), which clusters individuals who exhibit similar patterns in SEB dimensional scores on a Likert-type scale, has identified five predominant profiles: sophisticated, intermediate, absolutistic, multiplistic, and evidence-based (see Table 1 for details).

**Table 1:** Description of predominant SEB profiles.

Profile	Description
Sophisticated	Individuals in this profile exhibit well-developed and balanced epistemic beliefs across all dimensions, representing the highest level of sophistication in the given context.
Absolutistic	This profile includes individuals who score comparatively low and balanced across all dimensions, showing more disagreement with adequate epistemic belief statements than their peers.
Intermediate	Individuals in this profile score close to the mean on all dimensions in a balanced manner, indicating a less differentiated profile. They are reluctant to take a particular epistemic position.
Evidence-based	Individuals in this non-linear profile strongly emphasize the importance of experimentation and empirical data, scoring high on the development and justification dimensions while scoring low on source and certainty dimensions. This profile represents a focus on the evolving nature of science.
Multiplistic	This non-linear profile represents individuals who believe that many can contribute to knowledge generation but underestimate the importance of supporting knowledge with evidence, scoring high on source and certainty dimensions while low on development and justification dimensions.

Research has consistently shown that students with more sophisticated SEB tend to exhibit higher academic motivation and achievement [[24], [25], [26]]. Conversely, less sophisticated belief profiles, such as intermediate, absolutistic, or multiplistic, often correlate with lower academic outcomes [27]. In essence, individuals with a more nuanced understanding of the tentativeness, dynamics, and complexity of scientific knowledge typically fare better in educational settings.

However, a glaring gap in the literature is the exclusive focus on student populations. To date, no study has ventured into exploring the SEB of adults. Our research aims to bridge this void. We posit that transitioning the exploration of SEB from student cohorts to adult populations is not only feasible but also imperative. Adults, too, should exhibit varying levels of SEB. Given that a significant portion of the adult population in China might not have had access to higher education due to the country's historical developmental trajectory, delving into their SEB becomes even more salient. By pioneering this exploration, our study not only expands the horizons of existing literature but also underscores the profound implications of understanding SEB across different age demographics.

TCM and Western medicine exhibit foundational differences, with the former deeply rooted in ancient philosophical principles and the latter grounded in evidence-based approaches and scientific methodologies. This foundational divergence can pose challenges, especially in accurately diagnosing diseases using the principles of pathology or anatomy, which are fundamental in Western medicine. The absence of rigorous scientific evidence supporting the efficacy and safety of TCM has been a significant barrier to its acceptance in modern societies [28]. However, the allure of TCM lies in its individualized treatments, tailored to address the unique needs and conditions of each patient, which has sustained its popularity. This personalized approach, intrinsic to TCM, offers a unique perspective on healing and wellness, appealing to those seeking alternative or complementary therapies. In this context, individuals with a more sophisticated SEB profile, who acknowledge the evolving nature of science, may be more receptive to TCM. These individuals are likely to appreciate the diversity in medical paradigms and are less prone to dismiss TCM solely because it does not conform to the temporally prevailing scientific standards of Western medicine. Consequently, this study, which aims to explore the interplay between SEB profiles and TCM acceptance, could provide valuable insights into the integration of diverse medical paradigms, fostering a more inclusive and holistic approach to healthcare.

### 1.3 Pediatric TCM

In recent years, pediatric TCM has seen notable advancements in China, attributed to both developments in TCM methodologies and the concerted efforts of practitioners. This branch of TCM, focusing on holistic and preventative care tailored to children through syndrome differentiation [29,30], holds a pivotal role in addressing childhood illnesses and enhancing personalized clinical management [31].

The state's support and promotion have facilitated the standardization of pediatric TCM [32], enabling it to yield positive outcomes for a variety of pediatric conditions, including those for which Western medicine may lack effective treatments or clear mechanisms, such as anorexia [33], malnutrition [34], diarrhea [35], constipation [36], asthma [[37], [38], [39], [40], [41], [42]], and other chronic diseases [43]. Pediatric TCM stands out particularly in treating nephrotic syndrome in children, adhering to modern safety standards, and minimizing the adverse effects of medications on children's growth and development [44].

Despite the advancements and positive outcomes associated with pediatric TCM, skepticism regarding its efficacy persists, with some individuals questioning whether the claims are overstated. This skepticism is particularly pronounced when guardians are selecting treatments for children, as the stakes are perceived to be higher compared to choosing treatments for adults. Given that an estimated 8 % of children and adolescents worldwide experience chronic diseases, which can lead to a range of social, emotional, developmental, and psychological challenges [45], the decision-making process for their treatment is fraught with heightened responsibility and caution. Guardians' involvement and discerning choices in managing diseases and selecting treatments for their children underscore the need for a thorough examination of the acceptance of pediatric TCM.

The focus on pediatric TCM rather than other branches of TCM in this study is deliberate and significant. The choice of treatment for children is often approached with greater caution by guardians, reflecting a more genuine and discerning acceptance level. If guardians are willing to opt for TCM for their children, it is highly likely they would do the same for themselves, but the converse may not hold true. Therefore, examining the acceptance of pediatric TCM can provide a more accurate reflection of the underlying attitudes and genuine acceptance levels towards TCM as a whole.

### 1.4 The present study

In this pioneering research, our primary focus was to unravel the intricate relationship between SEB and the acceptance of pediatric TCM. Adopting a novel multigroup path analysis, we segmented the participants (parents) into distinct subgroups based on their SEB profiles, employing a person-centered methodology. This innovative approach allowed us to delve deeper into the variations among these profiles within the UTAUT model's context concerning pediatric TCM. Our overarching objective was to illuminate the intricate connections between scientific literacy and TCM acceptability, thereby bridging the gap between age-old cultural medical practices and contemporary scientific paradigms. This pioneering endeavor seeks to redefine the discourse on traditional medical practices in the light of modern scientific comprehension.

## 2 Methods

### 2.1 Participants and procedure

The valid sample comprised 1016 Chinese parents, with 72.64 % being mothers. Regarding age distribution, 53.01 % were aged 30–39 years, 29.22 % were aged 40–49 years, and 12.93 % were younger than 30 years old. The majority of participants reported having two children (52.56 %). Ethnically, the vast majority identified as Han (96.56 %). Geographically, most were from Guangdong (53.15 %), followed by Hunan (9.94 %) and Jiangsu (3.64 %). Additionally, a significant portion (81.89 %) resided in urban areas. In terms of education, over half (56.00 %) held a bachelor's degree, while others had a vocational college education (15.94 %) or a master's degree (9.06 %).

The study received ethical approval from the Human Research Ethics Committee for Non-Clinical Faculties at the Center for Linguistics and Applied Linguistics, Guangdong University of Foreign Studies (protocol number: CLAL-202212-002; date of approval: December 3, 2022). Data collection began in July 2023 and ended in September 2023. Utilizing a convenience sampling method, participants were enlisted with the collaboration of several school headmasters spanning kindergartens, primary schools, and secondary schools. This approach ensured that all respondents were parents of children, guaranteeing a heightened sensitivity to the efficacy and safety aspects of pediatric TCM. The survey was disseminated through online platforms of Credamo (https://www.credamo.com/home.html), DiggMind (http://digme.cn/), and Wenjuanxing (https://www.wjx.cn/). All participants voluntarily completed the questionnaire, being fully informed about its purpose and content. They were made aware that they could withdraw from the survey at any stage if they felt uncomfortable. Electronic informed consent was obtained from all participants. Upon completion of the questionnaire, their responses were automatically screened by the platform using one discriminative item (responses with incorrect answers to the item “Please select ‘Strongly disagree’ on this item” were deemed invalid) and the duration of completion (the ones that took less than 2 min were also considered invalid). Participants who passed this initial screening received a small monetary reward in Chinese Yuan as a token of appreciation, distributed randomly. As a result, the platforms determined a total of 1176 questionnaires valid and restricted our access to any that were invalid. Subsequently, researchers conducted a thorough evaluation of the response quality of the 1176 collected questionnaires and further excluded 160 entries from the dataset for various reasons, including 52 entries with lengthy strings of invariant responses, 39 entries with obviously false information about participants' ages as parents, and 69 entries with identical responses submitted from different IP addresses at nearly the same time. In the end, a total of 1016 questionnaires were utilized for the formal analyses.

### 2.2 Measures

#### 2.2.1 UTAUT constructs

The six constructs of the UTAUT model pertaining to pediatric TCM were gauged using scales we developed ourselves. This was achieved by referencing the subfactors of the UTAUT scale formulated by Venkatesh et al. [11] and adapting them to fit the context of pediatric TCM among Chinese parents. Four items were crafted for each subscale. Examples include: “Traditional Chinese medicine has shown commendable performance in the recovery of children's illnesses” (performance expectancy), “Important people around me (such as spouses, close friends) believe that children should receive traditional Chinese medicine treatment when they are sick” (social influence), “It's convenient to consult traditional Chinese medicine when my child is ill” (facilitating condition), “I'm concerned about the safety risks of using traditional Chinese medicine” (risk awareness), “If my child falls sick, I would opt for traditional Chinese medicine treatment” (usage intention), and “My child frequently uses traditional Chinese medical treatments or consumes herbal medicine” (usage behavior). Group discussions were conducted to ensure the content validity of these items. Participants rated each item on a 6-point Likert scale, where 1 point signifies “Strongly disagree” and 6 points indicate “Strongly agree."

#### 2.2.2 SEB

SEB were assessed using a modified version of the 26-item instrument crafted by Conley et al. [23]. To streamline the questionnaire, we selected four items from each dimension. Representative items include: “Everybody has to believe what scientists say” (source), “All questions in science have one right answer” (certainty), “The ideas in science books sometimes change” (development), and “It is good to repeat experiments to confirm your findings” (justification). Given that our participants were Chinese parents, we translated the scales into Chinese and made minor adjustments to the wording for clarity. Items were rated on a 6-point Likert scale, with 1 indicating “Strongly disagree” and 6 denoting “Strongly agree.” The source and certainty scales were recoded in such a way that higher scores now represent a more mature understanding of the respective SEB.

#### 2.2.3 Covariates

Among the demographic information collected, gender, grade, and education level were included as covariates according to previous research [[7], [8], [9], [10]]. For gender, males were coded as 1 and females as 2. For age, individuals younger than 30 years were coded as 1, those aged 30–40 years as 2, those between 40 and 50 years as 3, and those older than 50 years as 4. For education level, junior high school and below were coded as 1, technical secondary school as 2, high school as 3, technical college as 4, bachelor's degree as 5, master's degree as 6, and doctoral degree as 7. These three variables can be considered continuous variables when entering into statistical analysis.

### 2.3 Data analyses

The data analysis process consisted of four main steps. First, a preliminary examination was conducted to ensure the data's quality and suitability for further analysis. During this phase, descriptive statistics were computed, and reliability and validity tests were performed. Second, we employed k-means cluster analysis, a method that partitions the sample into “k" clusters by minimizing within-cluster variance and maximizing between-cluster variance. The third step involved identifying potential significant covariates. Chi-square analyses were conducted to examine the relationships between SEB clusters and the three covariates, identifying significant covariates. Subsequently, with these significant covariates controlled, analyses of covariance (ANCOVA) were conducted on the UTAUT variables to assess subgroup disparities. Regression analyses were then separately performed on the UTAUT constructs against these significant covariates, with the residuals saved for subsequent path analyses. Finally, a multigroup path analysis was executed to explore the relationships between the UTAUT constructs across the different SEB profiles. This analysis helped us discern variations in the paths between constructs across the identified clusters, enabling comparisons of the strength and direction of these relationships within each cluster. These insights informed our understanding of how different belief profiles might influence TCM acceptance. All analytical procedures, except for the multigroup path analysis conducted using AMOS 25, were performed using Statistical Product and Service Solutions software (SPSSAU; https://spssau.com/index.html).

## 3 Results

### 3.1 Preliminary analysis

Prior to the main statistical analyses, preliminary evaluations were conducted to examine the measurement properties and descriptive statistics of the variables. As depicted in Table 2, all constructs demonstrated robust reliability, with Cronbach's alpha values exceeding 0.7 and composite reliability values surpassing 0.8. Convergent validity was affirmed with average variance extracted (AVE) values exceeding 0.5 for all factors. Discriminant validity was supported as the square root of AVE for each construct was greater than its inter-construct correlations. The structural validity of the measurement model also indicated an acceptable fit. Using Harman's single-factor test, the amount of variance explained by the first factor was 35.24 %, which was less than the threshold value of 40 %, indicating that common method bias was not a serious concern. These outcomes confirmed that our measures were both reliable and valid.

**Table 2:** Descriptives, correlation, reliability, and validity of the constructs.

	PE	SI	RA	FC	UI	UB	Sou	Cer	Dev	Jus
PE	**0.80**									
SI	0.75***	**0.82**								
RA	−0.44***	−0.41***	**0.80**							
FC	0.64***	0.73***	−0.40***	**0.83**						
UI	0.74***	0.81***	−0.49***	0.71***	**0.85**					
UB	0.64***	0.76***	−0.42***	0.72***	0.83***	**0.86**				
Sou	−0.14***	−0.21***	−0.02	−0.21***	−0.17***	−0.21***	**0.73**			
Cer	−0.15***	−0.22***	0.01	−0.26***	−0.20***	−0.26***	0.66***	**0.71**		
Dev	0.23***	0.24***	−0.01	0.21***	0.26***	0.20***	0.04	0.09**	**0.68**	
Jus	0.33***	0.36***	−0.17***	0.33***	0.39***	0.31***	−0.14***	−0.05	0.61***	**0.82**
M	4.81	4.54	2.93	4.25	4.59	4.19	3.35	3.74	4.63	4.86
SD	0.9	0.95	1.08	1.09	1.01	1.16	1.04	0.98	0.76	0.77
α	0.87	0.89	0.87	0.89	0.91	0.92	0.82	0.77	0.77	0.89
AVE	0.64	0.68	0.63	0.68	0.73	0.73	0.53	0.5	0.46	0.68
CR	0.87	0.89	0.87	0.89	0.92	0.92	0.82	0.79	0.77	0.89

Notes: **p < 0.01, ***p < 0.001. The bold and underlined numbers on the diagonal represent the square root of AVE values. The model fit for the 10-factor confirmatory factor analysis is as follows: χ^2^/df = 2312.508/695 = 3.327, p < 0.001, RMSEA = 0.048 < 0.08, SRMR = 0.048 < 0.08, CFI = 0.943 > 0.9, TLI = 0.936 > 0.9.

Abbreviations: PE, performance expectancy; SI, social influence; RA, risk awareness; FC, facilitating condition; UI, usage intention; UB, usage behavior; Sou, source; Cer, certainty; Dev, development; Jus, justification.

Regarding the descriptive statistics and correlation matrix, the six UTAUT variables displayed moderate to strong correlations. Performance expectancy, social influence, facilitating conditions, usage intention, and behavior showed positive interrelationships, while risk awareness was negatively associated. This pattern was consistent with the hypothesized relationships in the research model.

The correlations between the SEB dimensions provided nuanced insights. Source and certainty showed a strong positive correlation, suggesting those viewing scientific knowledge as originating from authorities also believed that such knowledge was fixed and certain. Similarly, development and justification were strongly positively correlated, indicating those who believed scientific knowledge changes over time also tended to value evidence-based justification. However, there seemed to be a distinction between the source and certainty dimensions and the development and justification dimensions. These correlations illuminated the complex dynamics between epistemic belief dimensions underlying individuals' scientific perspectives.

Interestingly, while the source and certainty dimensions were negatively related to TCM usage, the development and justification dimensions exerted positive influence. It demonstrates that individuals recognizing science as an evolving process grounded in emerging evidence were more open to TCM. Conversely, those clinging rigidly to scientific facts from authorities as immutable truth also exhibited higher inclination towards TCM. This highlights the nonlinear relationships between scientific literacy and TCM acceptance. These intriguing findings provided a basis for testing the UTAUT model and exploring multigroup differences based on SEB profiles.

### 3.2 K-means cluster analysis

**Fig. 2:**
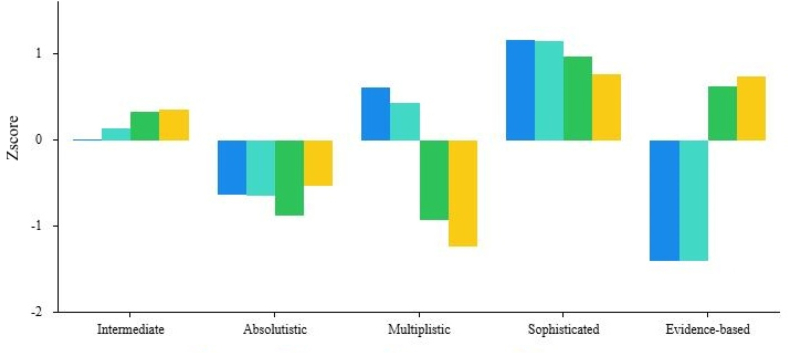
Membership of SEB profiles.

To discern distinct SEB profiles among the participants, we employed extensive k-means cluster analyses, ranging from k = 2 to k = 8, as detailed in Table 3. We not only based our selection criteria on the relatively lower value of the sum of squares error and the higher value of the silhouette coefficient, but took into account the gap statistics between adjacent solutions we well, while also ensuring a balanced size (not smaller than 10 % of the total sample) for each subgroup and emphasizing the theoretical interpretability of the clusters, which eventually steered us towards the k = 5 solution as optimal. Drawing from profile naming conventions and descriptions in previous research on student samples (see Table 1 and Fig. 2), the five clusters identified were labeled as follows: “intermediate”, “absolutistic”, “multiplistic”, “sophisticated”, and “evidence-based”, respectively.

**Table 3:** Solutions of extensive k-means cluster analyses.

Solution	SSE	SC	Cluster 1	Cluster 2	Cluster 3	Cluster 4	Cluster 5	Cluster 6	Cluster 7	Cluster 8
K = 2	2985.89	0.263	45.28 %	54.72 %						
K = 3	2104.76	0.365	35.04 %	28.05 %	36.91 %					
K = 4	1869.56	0.386	20.37 %	28.44 %	19.49 %	31.69 %				
K = 5	1637.60	0.422	30.22 %	19.78 %	19.39 %	16.73 %	13.88 %			
K = 6	1489.24	0.444	21.95 %	19.00 %	17.81 %	18.11 %	8.86 %	14.27 %		
K = 7	1385.65	0.459	21.46 %	3.44 %	18.50 %	18.60 %	8.76 %	13.68 %	15.55 %	
K = 8	1300.33	0.475	19.00 %	3.44 %	16.34 %	13.19 %	8.27 %	12.60 %	15.45 %	11.71 %

Notes: The solution with k = 5, highlighted in the bold row, was the one we ultimately chose.

Abbreviations: SSE, sum of squares error; SC, silhouette coefficient.

Chi-square analyses indicated significant associations between the three covariates and SEB profiles. Specifically, for gender (χ^2^ = 12.95, p = 0.012), males tended to fall into the intermediate category, while females tended to be in the absolutistic and multiplistic categories. Regarding age (χ^2^ = 66.44, p < 0.001), individuals younger than 30 years tended to fall into the sophisticated and intermediate categories, while those aged 40–50 years tended to fall into the evidence-based and absolutistic categories. In terms of education level (χ^2^ = 102.68, p < 0.001), individuals with a bachelor's degree tended to fall into the Sophisticated and Intermediate categories. Hence, the subsequent analyses will need to control for these three covariate.

A deeper exploration of the six UTAUT variables across these profiles using ANCOVA, as presented in Table 4, revealed significant disparities. Notably, the evidence-based profile consistently registered the highest mean scores across performance expectancy, social influence, facilitating condition, as well as usage intention and behavior, indicating a pronounced willingness towards accepting pediatric TCM despite relatively higher risk awareness. On the contrary, the multiplistic profile demonstrated extreme reservations, particularly reflected in the highest score of the risk awareness dimension, suggesting heightened apprehensions related to TCM. Interestingly, among the three balanced belief profiles, the intermediate scored significantly higher than both the absolutistic and the sophisticated on five of the six variables (except risk awareness), but there was no significant difference between the absolutistic and the sophisticated. This suggests that individuals with a high level of scientific literacy do not blindly believe in TCM, while those with a more limited scientific foundation struggle to discern the merits of TCM. These findings underscore the intricate relationship between an individual's scientific beliefs and their disposition towards TCM, highlighting the multifaceted determinants that influence TCM acceptance.

**Table 4:** Comparison of UTAUT variables across SEB profiles.

	Cluster 1: Intermediate (n = 307)	Cluster 2: Absolutistic (n = 201)	Cluster 3: Multiplistic (n = 197)	Cluster 4: Sophisticated (n = 170)	Cluster 5: Evidence-based (n = 141)	F	η_p_^2^	Post hoc comparison
PE	4.96 ± 0.79	4.73 ± 0.89	4.34 ± 0.89	4.89 ± 0.92	5.17 ± 0.84	15.18***	0.10	5 > 1>2 > 3; 5 > 4>3
SI	4.67 ± 0.84	4.48 ± 0.84	3.99 ± 0.89	4.56 ± 1.07	5.06 ± 0.88	19.01***	0.12	5 > 1>2 > 3; 5 > 4>3
RA	2.85 ± 1.00	2.80 ± 1.04	3.19 ± 0.85	2.81 ± 1.14	3.05 ± 1.40	3.52**	0.02	35 > 124
FC	4.36 ± 1.06	4.25 ± 0.90	3.63 ± 1.01	4.24 ± 1.15	4.87 ± 0.98	21.33***	0.13	5 > 142>3
UI	4.83 ± 0.86	4.48 ± 0.92	3.91 ± 0.95	4.66 ± 1.14	5.09 ± 0.85	25.48***	0.15	5 > 1>2 > 3; 5 > 4>3
UB	4.41 ± 1.03	4.17 ± 0.98	3.54 ± 1.03	4.07 ± 1.38	4.77 ± 1.09	19.56***	0.12	5 > 1>24 > 3

Notes: **p < 0.01, ***p < 0.001. For the covariates, gender had no significant influence on the UTAUT constructs; age had a significant influence on FC (F = 4.77, p = 0.029), UI (F = 5.17, p = 0.023), and UB (F = 6.82, p = 0.009); education level had a significant influence on FC (F = 9.90, p = 0.002) and RA (F = 3.99, p = 0.046). Abbreviations: PE, performance expectancy; SI, social influence; RA, risk awareness; FC, facilitating condition; UI, usage intention; UB, usage behavior.

### 3.3 Multigroup path analysis

**Fig. 3:**
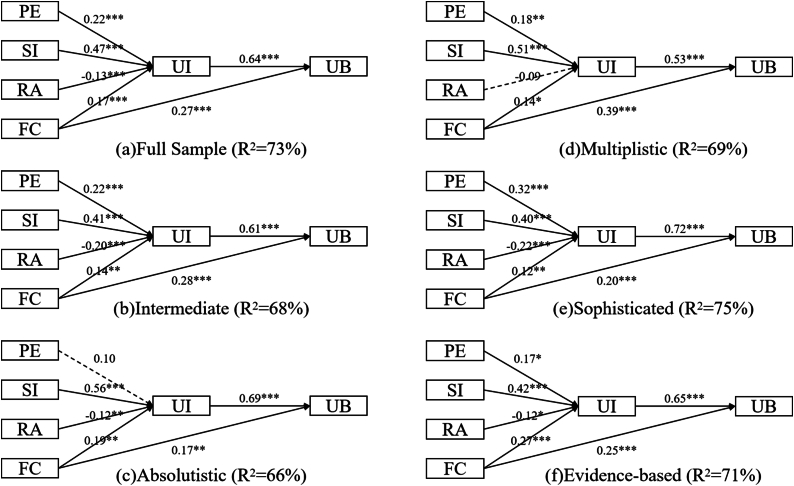
Results of multigroup path analysis. *p < 0.05, **p < 0.01, ***p < 0.001. PE = performance expectancy, SI = social influence, RA = risk awareness, FC = facilitating condition, UI = usage intention, UB = usage behavior. Intercorrelations between the four exogenous variables were omitted for clarity.

Following the identification of distinct SEB profiles, we initiated a multigroup path analysis to explore the differential effects of the UTAUT variables across these profiles. Prior to this, we regressed the UTAUT constructs against the three significant covariates and saved the residuals as variable values for the UTAUT constructs in the subsequent multigroup path analysis. Besides, we also examined measurement invariance across subgroups, and the indices demonstrated acceptable fit: for configural invariance, χ^2^ = 3800.758, df = 1675, CFI = 0.903, RMSEA = 0.079; for metric invariance, χ^2^ = 3923.750, df = 1763, CFI = 0.902, RMSEA = 0.078; for scalar invariance, χ^2^ = 4164.557, df = 1851, CFI = 0.895, RMSEA = 0.078. The formal multigroup path analysis, as illustrated in Fig. 3 and Table 5, revealed a significant moderating effect of SEB profiles on the UTAUT path model.

**Table 5:** Indirect effect analyses.

Group	Indirect effect	Effect	Bootstrap 5000 times 95 % CI		p
			Lower	Upper	
Full sample	PE→UI→UB	0.18	0.13	0.23	0.000***
	SI→UI→UB	0.37	0.3	0.44	0.000***
	RA→UI→UB	−0.09	−0.12	−0.06	0.000***
	FC→UI→UB	0.12	0.08	0.16	0.000***
Intermediate	PE→UI→UB	0.18	0.09	0.26	0.000***
	SI→UI→UB	0.31	0.17	0.47	0.000***
	RA→UI→UB	−0.13	−0.21	−0.05	0.000***
	FC→UI→UB	0.08	0.01	0.15	0.027*
Absolutistic	PE→UI→UB	0.07	−0.01	0.17	0.096
	SI→UI→UB	0.44	0.29	0.58	0.000***
	RA→UI→UB	−0.07	−0.14	−0.01	0.034*
	FC→UI→UB	0.14	0.05	0.25	0.002**
Multiplistic	PE→UI→UB	0.11	0.02	0.21	0.020*
	SI→UI→UB	0.31	0.18	0.45	0.000***
	RA→UI→UB	−0.06	−0.14	0.02	0.136
	FC→UI→UB	0.07	0	0.16	0.057
Sophisticated	PE→UI→UB	0.35	0.22	0.5	0.000***
	SI→UI→UB	0.37	0.25	0.49	0.000***
	RA→UI→UB	−0.19	−0.29	−0.11	0.000***
	FC→UI→UB	0.11	0.01	0.22	0.029*
Evidence-based	PE→UI→UB	0.15	−0.03	0.33	0.108
	SI→UI→UB	0.33	0.19	0.48	0.000***
	RA→UI→UB	−0.06	−0.12	−0.01	0.018*
	FC→UI→UB	0.19	0.07	0.34	0.001**

Notes: *p < 0.05, **p < 0.01, ***p < 0.001.

Abbreviations: PE, performance expectancy; SI, social influence; RA, risk awareness; FC, facilitating condition; UI, usage intention; UB, usage behavior.

For the entire sample, the UTAUT model exhibited strong predictive power. Among the four exogenous independent variables, social influence had the most significant positive impact on usage intention, followed by performance expectancy, suggesting that parents' acceptance of pediatric TCM was primarily driven by recommendations from others, and secondarily by their own perceptions of its effectiveness. Facilitating conditions also positively influenced usage intention but exerted a more pronounced impact on usage behavior, underscoring the significance of further improving the convenience of pediatric TCM medical services. Risk awareness, on the other hand, had a negative but the smallest influence on usage intention, suggesting that the majority of participants were not overly concerned about the safety risks of TCM.

The sophisticated and intermediate profiles mirrored the UTAUT patterns observed in the full sample model, with all four independent factors being influential. Notably, the sophisticated model exhibited the strongest positive association between performance expectancy and usage intention among the six groups, highlighting the rational discernment characteristic of this profile.

Intriguingly, both the absolutistic and evidence-based subgroups exhibited a nonsignificant mediating pathway from performance expectancy to usage behavior via usage intention. However, the underlying mechanisms for this phenomenon might differ markedly between the two subgroups. For the absolutistic profile, the lack of scientific literacy might hinder them from evaluating the efficacy of TCM. Conversely, in the evidence-based profile, the nonsignificant mediating pathway can be attributed to a unanimous consensus within the group about TCM's effectiveness, making this variable less influential within this specific subgroup. Similarly, this rationale can be applied to understand why, in the multiplistic profile, risk awareness and facilitating conditions did not have a significant influence on usage behavior via usage intention. The uniform high reservations about the safety of TCM and the overwhelming belief that TCM facilitating conditions were too difficult to access among this subgroup rendered these two variables less impactful in their attitudes towards TCM.

In summary, while there were overarching trends consistent across profiles, such as the positive influence of social influence on usage intention, there were also profile-specific nuances. These differences underline the importance of considering individual SEB when examining TCM acceptability.

## 4 Discussions

The present study represents a pioneering effort as the first empirical exploration of the complex relationship between scientific literacy and TCM acceptability. Our study introduced a series of novel approaches that bring fresh perspectives to the research landscape. Conceptually, we used SEB as an indicator to reflect fundamental aspects of scientific literacy. Methodologically, we employed multigroup path analysis to integrate SEB as a moderator into the UTAUT model. In terms of implementation, we strategically focused on pediatric patients and their parents to capture authentic attitudes towards TCM. Despite certain inherent limitations, our findings illuminate the complex dynamics that shape individual perceptions and behaviors towards TCM. This discussion section explores the implications of our findings, highlighting both the broad trends and the nuanced distinctions within each SEB profile. It also identifies areas for future research that could address the limitations of the current study and further our understanding of this complex topic.

### 4.1 UTAUT model of TCM: A novel application

In a pioneering initiative, we introduced the UTAUT model to the realm of TCM, a domain where it had not been previously explored. The congruence between the observed correlations among the UTAUT variables and the hypothesized relationships reinforced the applicability and relevance of the UTAUT framework in understanding TCM acceptance. This novel application not only offers fresh insights but also underscores the versatility of the UTAUT model.

According to our results, the UTAUT model demonstrated its robustness in explaining Chinese people's acceptance of TCM and revealing the relative significance of various determinants. Of the four primary independent variables, social influence from trusted sources emerged as the most potent predictor of TCM attitude, highlighting the power of word-of-mouth and referrals in TCM promotion [44]. Closely following, personal convictions about TCM's efficacy also carried substantial weight, underlining the importance of establishing and bolstering TCM's credibility [46]. Meanwhile, facilitating conditions, though positively influencing usage intention, had an even more considerable effect on actual usage behavior, suggesting that enhanced accessibility and convenience of TCM services could further drive its adoption [47]. On the other hand, risk awareness, albeit negatively correlated, exerted a comparatively muted influence on usage intention, indicating that for the majority, safety concerns related to TCM were not a primary deterrent.

Overall, our findings underscore a remarkably high level of endorsement, acceptance, and trust among the Chinese population towards TCM [48]. Moreover, we have faith in the reliability of these results, given our astute choice of measuring parents' acceptance of pediatric TCM, which offers a more genuine reflection of actual attitudes while mitigating potential biases such as social desirability effects [49]. Nonetheless, a meticulous examination of the TCM-UTAUT model across different scientific literacy profiles unveiled even more intriguing variations and insights, which are discussed in the subsequent sections.

### 4.2 SEB profiles and TCM acceptance

With its distinct origins diverging from modern medicine, TCM has perennially been a subject of contention within the contemporary scientific paradigm. A prevailing stereotype has long suggested that those who embrace TCM typically possess a lower scientific literacy [9]. However, a recent landmark study has painted a different picture, indicating that the primary proponents of TCM are, in fact, younger individuals with higher educational backgrounds [7]. This challenges the conventional narrative and underscores the pressing need to re-evaluate the relationship between scientific literacy and TCM acceptance. In light of this, our research embarked on an unprecedented empirical investigation into this very issue, aiming to shed light on the intricate dynamics at play.

We believe the choice of SEB as a central indicator of scientific literacy is innovative. This is because its emphasis on understanding scientific discoveries and inquiries can potentially carve out an appropriate niche for TCM within the modern scientific framework. Yet, existing literature on SEB has predominantly focused on student populations [50]. Venturing into uncharted territory, our study boldly extended this focus to the adult population. In doing so, we were able to identify the same five profiles that previous research has frequently observed within student populations: the “intermediate” with average scores across all dimensions; the “absolutistic” with uniformly low scores; the “sophisticated” with consistently high scores; the “multiplistic” excelling in source and certainty but lagging in development and justification; and the “evidence-based” showing the opposite pattern of the multiplistic [50]. This not only validates the universality of these profiles, but also highlights the broader applicability of SEB across age groups.

Each of the five profiles presented distinct UTAUT patterns regarding TCM acceptance. The multiplistic profile was the least accepting of TCM. Characterized by skepticism, this subgroup tends to question and deny almost everything. While it is commendable that they do not blindly follow authority and acknowledge the potential flaws in science [51], their skepticism extends to the point of even overshadowing the improving nature of scientific research and downplaying objective evidence [52]. This extreme negativity devoid of balanced evidentiary assessment exaggerates TCM's side effects and hinders their adoption.

Contrastingly, the evidence-based profile demonstrates the most favorable stance towards TCM. Their understanding of science as a continually evolving field suggests that the current lack of full scientific endorsement for TCM does not negate its future potential [27]. Their emphasis on evidence acknowledges TCM's effectiveness, also backed by widespread practical use. However, a potential pitfall for this subgroup is that their overreliance on authority and the deficiency of awareness that knowledge can be fallible signify their excessive trust in TCM's performance may be tinged with a degree of blind faith.

The absolutistic, intermediate, and sophisticated profiles have moderate acceptance levels of TCM. Intriguingly, among these, the intermediate group, with its mid-level scientific literacy, shows the highest endorsement of TCM, whereas the absolutistic and sophisticated subgroups, representing the lowest and highest ends of the scientific literacy spectrum respectively, surprisingly have a similar level of TCM recognition. This nonlinear relationship suggests that while individuals with high scientific literacy approach TCM with a critical and informed perspective, avoiding blind adherence, those with limited scientific understanding might overlook the rational and evidence-based facets of TCM, potentially leading to misconceptions or uninformed decisions.

In summation, our study offers a nuanced understanding of the relationship between SEB and TCM acceptance. Contrary to prevailing stereotypes, TCM's primary proponents are not necessarily limited to those with lower scientific literacy. Instead, its acceptance spans across various profiles of scientific understanding, from the skeptical multiplistic to the evidence-based believers. Our findings underline the importance of recognizing the multifaceted nature of scientific literacy and its implications for TCM acceptance. The study reveals that while high scientific literacy promotes a balanced and critical view of TCM, a lack thereof might lead to either blind adherence or unwarranted skepticism. This research, by extending the study of SEB to adults and correlating it with TCM acceptance, offers invaluable insights for both the scientific and traditional medical communities, emphasizing the need for informed decision-making and bridging the gap between modern science and traditional practices.

### 4.3 Practical implications

Now that TCM culture is experiencing unprecedented development opportunities as people re-examine their perspectives on health. The holistic approach, emphasis on prevention, and personalized treatment advocated by TCM are gaining increasing recognition. Simultaneously, TCM has demonstrated unique advantages in the prevention and treatment of various diseases [48]. The World Health Organization has continually called upon countries to prioritize the protection and inheritance of their local traditional medicine [53]. Against this backdrop, promoting TCM culture and fostering a broader understanding and acceptance of TCM practices are crucial for advancing the TCM field, constructing a diverse and coordinated healthcare system, and hold significant implications.

Based on the findings of this study, there are several practical implications that can be drawn to promote TCM culture. Firstly, our study underscores the significant impact of social influence on the acceptance and adoption of pediatric TCM. To leverage this influence, targeted promotional campaigns, educational initiatives, and endorsements from trusted sources should be utilized. By harnessing social networks, word-of-mouth, and peer recommendations, awareness and positive perceptions of TCM can be enhanced among the public. Secondly, the study reveals a nuanced relationship between scientific literacy and TCM acceptance, suggesting the need to address biases and misconceptions. Efforts should focus on enhancing the general population's scientific literacy, emphasizing the dynamic nature of scientific knowledge. Educational programs, public outreach initiatives, and science communication strategies can play a pivotal role in fostering a nuanced understanding of scientific inquiry and the potential of traditional practices to complement modern medicine. Lastly, to enhance the acceptance and integration of TCM into mainstream healthcare, its scientific credibility must be strengthened. This can be achieved through rigorous, evidence-based research and validation studies adhering to evidence-based medicine principles [54]. Additionally, stringent quality control measures, regulatory frameworks, and monitoring systems should be implemented to ensure consistent quality and safety of TCM products and practices. These measures can mitigate potential risks and build trust among the public and healthcare professionals.

### 4.4 Limitations and future directions

Inevitably, our study is not without its limitations. A primary constraint lies in our sampling strategy. The convenience sample, primarily drawn from southern cities of China, may not be sufficiently representative. This raises concerns about the generalizability of our findings to broader demographics, especially those from different eco-cultural backgrounds or other regions of China. Such a localized sample might inadvertently introduce regional biases, given the diverse cultural and medical practices across China. Besides, the intentional choice of parents as participants for their potential to offer authentic insights could also introduce bias. For one, ethical regulations prohibited us from gathering parents' more detailed demographic information, such as occupation and salary, potentially leaving out important covariates. This limitation underscores the need for larger-scale and more comprehensive future research on TCM acceptance.

Second, the cross-sectional design of our research offers a limited perspective, capturing only a snapshot of the intricate relationship between scientific literacy and TCM acceptance at a specific point in time. This design inherently lacks the ability to track changes and evolutions in attitudes and beliefs. A longitudinal approach would be more illuminating in this regard, allowing for a dynamic understanding of how perceptions and attitudes towards TCM and scientific literacy evolve over time, especially considering the rapidly changing landscapes of both TCM practices and public understanding of science [55].

Third, while we assessed the general acceptance of TCM, we did not delve into the depth of participants' understanding of various TCM practices or their specific reasons for acceptance or skepticism. External influences, such as media portrayals, government policies, or recent advancements in TCM research, might have also shaped participants' responses, a factor not controlled for in our study. Additionally, different TCM treatments might elicit different attitudes, which should be explored in future studies. Furthermore, other aspects of scientific literacy beyond SEB might also influence TCM acceptance, a consideration that future research should also address.

Looking ahead, several avenues beckon further exploration. It would be enlightening to study similar dynamics across different age groups, cultural backgrounds, or even among healthcare professionals to paint a more comprehensive picture. Interventional studies that aim to enhance scientific literacy and then measure changes in TCM acceptance could provide a clearer understanding of causality. Delving deeper, qualitative methodologies, such as interviews or focus groups, could offer richer insights into the reasons behind individuals' stances on TCM. As TCM continues its trajectory of global recognition, research that explores its integration with modern medicine, and the role scientific literacy plays in this melding, is of paramount importance. Moreover, the framework and methodologies employed in our study could be adapted to explore other traditional medicinal practices, such as Ayurveda or African herbal medicine, to understand the broader interplay between scientific literacy and traditional medical systems.

In sum, our research lays foundational groundwork in an intriguing domain, but the landscape of unexplored questions remains vast. We are optimistic that our findings will serve as a catalyst for more nuanced and diverse investigations in the future.

## 5 Conclusion

The intricate relationship between scientific literacy and TCM acceptance has long been a subject of interest and debate. Our pioneering research has shed significant light on this relationship, offering a fresh perspective that challenges prevailing stereotypes and broadens our understanding of the dynamics at play.

Our study stands out as the first to empirically explore the nexus between SEB and pediatric TCM acceptance within a parent population. By identifying the same five profiles of SEB that have been observed in student populations, we have validated the universality of these profiles and underscored the broader applicability of these beliefs across age groups.

The findings reveal a complex landscape: while some profiles, like the evidence-based believers, show a strong inclination towards TCM, others, such as the multiplistic group, exhibit skepticism. Intriguingly, the nonlinear relationship between scientific literacy and TCM acceptance suggests that neither blind adherence nor unwarranted skepticism is exclusive to any particular level of scientific understanding.

Furthermore, the application of the UTAUT model in the realm of TCM has proven to be a novel and effective approach, revealing the multifaceted determinants that influence TCM acceptance. From the power of social influence to personal beliefs about TCM's efficacy, our research has highlighted the myriad factors that shape individuals' attitudes towards TCM.

In closing, our research underscores the importance of a holistic understanding of the interplay between scientific literacy and TCM acceptance. As the world continues to grapple with the integration of traditional and modern medical practices, studies like ours provide a foundation for informed discourse and decision-making. We hope that our findings will pave the way for further research in this domain, fostering a deeper understanding and appreciation of the rich tapestry of medical knowledge that spans cultures and epochs.

## Funding statement

This research was funded by grants from Xiaorong Luo's Renowned Expert Inheritance Studio of National Administration of Traditional Chinese Medicine (No.14GG2X02).

## Data availability statement

The data presented in this study are available upon reasonable request from the corresponding author. The data are not publicly available due to privacy or ethical restrictions.

## Institutional review board statement

The study was conducted in accordance with the Declaration of Helsinki, and approved by the Human Research Ethics Committee for Non-Clinical Faculties of the Center for Linguistics and Applied Linguistics, Guangdong University of Foreign Studies (protocol number: CLAL-202212-002; date of approval: December 3, 2022).

## Informed consent statement

Electronic informed consent was obtained from all subjects involved in the study.

## Consent for publication

Not applicable.

## CRediT authorship contribution statement

**Minrui Zhang:** Writing – original draft, Visualization, Resources, Investigation, Formal analysis, Conceptualization. **Aiyuan Cai:** Writing – original draft, Visualization, Validation, Software, Investigation, Formal analysis. **Kexin Jin:** Writing – original draft, Visualization, Validation, Software, Investigation, Formal analysis. **Jiaying Huang:** Visualization, Validation, Software, Resources. **Dan Li:** Writing – review & editing, Supervision, Resources, Project administration, Funding acquisition. **Meihui He:** Writing – review & editing, Validation, Software, Project administration, Formal analysis, Data curation, Conceptualization. **Ruixiang Gao:** Writing – review & editing, Project administration, Methodology, Formal analysis, Data curation, Conceptualization.

## Declaration of competing interest

The authors declare that they have no known competing financial interests or personal relationships that could have appeared to influence the work reported in this paper.
