# Supplementary material for: Scientific epistemology beliefs and acceptance of Traditional Chinese Medicine: A multigroup analysis based on the UTAUT model in Southern China
Source: Heliyon. 2024 Jun 17;10(12):e33136. doi: 10.1016/j.heliyon.2024.e33136 (PMC11252763; doi:10.1016/j.heliyon.2024.e33136)
Supplement: Multimedia component 1 [file mmc1.docx]

**Questionnaire Items and their Factor Loadings**

| **Unified Theory of Acceptance and Use of Technology** | |  |
| --- | --- | --- |
| *#效用期望* | *#Performance Expectancy* |  |
| 1. 中医儿科治疗疾病的临床效果明显。 | Traditional Chinese pediatric medicine shows significant clinical efficacy in treating diseases. | 1.000 |
| 1. 中医儿科对于治未病（预防疾病）具有重要作用。 | Traditional Chinese pediatric medicine plays a crucial role in disease prevention. | 1.037 |
| 1. 中医药在儿童疾病康复中有着不错的表现。 | Traditional Chinese medicine has shown commendable performance in the recovery of children's illnesses. | 0.970 |
| 1. 中医药能让我的孩子受益。 | Traditional Chinese medicine can benefit my child. | 1.062 |
| *#社会影响* | *#Social Influence* |  |
| 1. 我身边重要的人（如配偶、好友）认为儿童在生病时应当接受中医药治疗。 | Important people around me (such as spouses, close friends) believe that children should receive traditional Chinese medicine treatment when they are sick. | 1.000 |
| 1. 能够影响我行为的人（如老师、长辈）推荐我在孩子生病时让其使用中医药疗法。 | Individuals who can influence my behavior (such as teachers or elders) recommend using traditional Chinese medicine when my child is sick. | 0.965 |
| 1. 我重视其意见的人（如专家）认为中医儿科疗效显著。 | People whose opinions I value (such as experts) believe in the significant efficacy of traditional Chinese pediatric medicine. | 0.944 |
| 1. 总的来说，我周围的人支持我带我的孩子去看中医。 | Overall, the people around me support me in taking my child to see a traditional Chinese medicine practitioner. | 1.003 |
| *#便利条件* | *#Facilitating Condition* |  |
| 1. 孩子生病时看中医很方便。 | It's convenient to consult traditional Chinese medicine when my child is ill. | 1.000 |
| 1. 中医儿科疗法和药物价格便宜。 | Pediatric traditional Chinese medicine treatments and medications are affordable. | 0.871 |
| 1. 我能便捷地购买到中医药让我的孩子使用。 | I can easily purchase traditional Chinese medicine for my child's use. | 1.020 |
| 1. 我的孩子能享受到便利的中医药服务。 | My child can enjoy convenient traditional Chinese medicine services. | 1.062 |
| *#风险认知* | *#Risk Awareness* |  |
| 1. 我担心使用中医药具有安全风险。 | I'm concerned about the safety risks of using traditional Chinese medicine. | 1.000 |
| 1. 中医药治疗中的不良事件引发我的担忧。 | Adverse events during traditional Chinese medicine treatments raise my concerns. | 0.957 |
| 1. 我对于让孩子接受中医药治疗并不太放心。 | I am not entirely confident about letting my child undergo traditional Chinese medicine treatment. | 0.918 |
| 1. 如果要让我的孩子接受中医治疗，我会很谨慎。 | If considering traditional Chinese treatment for my child, I would be very cautious. | 0.816 |
| *#使用意愿* | *#Usage Intention* |  |
| 1. 我对中医儿科持积极的态度。 | I hold a positive attitude towards traditional Chinese pediatric medicine. | 1.000 |
| 1. 当朋友的孩子生病了，我会推荐他们看中医儿科。 | If a friend's child falls ill, I would recommend seeking treatment from a traditional Chinese pediatrician. | 1.350 |
| 1. 如果有可能，将来有需要时我会更多地带孩子看中医而非西医。 | If possible, in the future, I would prefer taking my child to see a traditional Chinese medicine practitioner rather than a Western one when needed. | 1.384 |
| 1. 如果我的孩子生病了，我会让其接受中医药治疗。 | If my child falls sick, I would opt for traditional Chinese medicine treatment. | 1.307 |
| *#使用行为* | *#Usage Behavior* |  |
| 1. 我的孩子经常使用中医疗法或吃中药。 | My child frequently uses traditional Chinese medical treatments or consumes herbal medicine. | 1.000 |
| 1. 我经常在生活中运用中医药保健理念和知识为我的孩子调理健康。 | I regularly apply traditional Chinese health preservation concepts and knowledge to manage my child's health. | 0.892 |
| 1. 孩子生病时，我更多带他（她）去看中医而不是西医。 | When my child is sick, I prefer taking them to a traditional Chinese medicine practitioner rather than a Western doctor. | 1.046 |
| 1. 中医养生保健已经融入了我的孩子的生活中。 | Traditional Chinese health preservation has become integrated into my child's life. | 0.996 |
| **Scientific Epistemic Beliefs** | |  |
| *#知识的来源* | *#Source* |  |
| 1. 每个人都必须相信科学家的说法。 | Everybody has to believe what scientists say. | 1.000 |
| 1. 只有科学家才能发现科学中的真理。 | Only scientists know for sure what is true in science. | 0.993 |
| 1. 如果你在一本科学书上读到某内容，你可以肯定这是真的。 | If you read something in a science book, you can be sure its true. | 0.954 |
| 1. 在科学领域，你必须相信科学书籍对事物的描述。 | In science, you have to believe what the science books say about stuff. | 0.951 |
| *#知识的确定性* | *#Certainty* |  |
| 1. 科学中的所有问题都有一个正确的答案。 | All questions in science have one right answer. | 1.000 |
| 1. 科学知识总是正确的。 | Scientific knowledge is always true. | 1.008 |
| 1. 一旦科学家们从实验中得出结果，这就是唯一的答案。 | Once scientists have a result from an experiment, that is the only answer. | 0.896 |
| 1. 科学家们总是对何为科学中的真理有一致的意见。 | Scientists always agree about what is true in science. | 0.422 |
| *#知识的发展* | *#Development* |  |
| 1. 今天科学界的一些看法与过去科学家的看法不同。 | Some ideas in science today are different than what scientists used to think. | 1.000 |
| 1. 科学书籍中的观点有时会改变。 | The ideas in science books sometimes change. | 1.103 |
| 1. 有些问题连科学家也无法回答。 | There are some questions that even scientists cannot answer. | 1.158 |
| 1. 科学界的看法有时会改变。 | Ideas in science sometimes change. | 1.274 |
| *#知识的正当性* | *#Justification* |  |
| 1. 科学实验的想法来自于对事物是如何运作的好奇和思考。 | Ideas about science experiments come from being curious and thinking about. | 1.000 |
| 1. 在科学研究中，科学家可以有不止一种方法来检验他们的想法。 | In science, there can be more than one way for scientists to test their ideas. | 1.009 |
| 1. 科学的一个重要部分是进行实验以对事物的工作原理提出新的想法。 | One important part of science is doing experiments to come up with new ideas about how things work. | 0.940 |
| 1. 多次尝试实验以确认你的发现是一件很好的事情。 | It is good to try experiments more than once to make sure of your findings. | 0.894 |
